# Visualizing Multi-Step Decision-Making at a Glance: Pairing Choose Your Own Adventure Style Simulated Cases with a Novel Mapping Framework

**DOI:** 10.1007/s40670-025-02505-6

**Published:** 2025-09-12

**Authors:** Caitlin D. Hanlon, Harry R. Goldberg, Stacy L. Cooper

**Affiliations:** 1 Biology Department, Quinnipiac University, 275 Mt Carmel Ave, Hamden, CT USA; 2 Department of Biomedical Engineering, Johns Hopkins University School of Medicine, Baltimore, MD USA; 3 Johns Hopkins University School of Medicine, Baltimore, MD USA

**Keywords:** Clinical reasoning, Medical education, Choose Your Own Adventure (CYOA), Decision-making, Dynamic mapping tools, Visualization

## Abstract

Clinical reasoning is a critical component of medical education, yet assessing decision-making in real-world scenarios remains challenging. Traditional assessment methods, such as multiple-choice questions, fail to replicate the dynamic nature of clinical problem-solving, where decisions build upon one another over time. Choose Your Own Adventure (CYOA) Case studies offer a promising alternative by simulating non-linear, multi-step decision-making. This study aimed to pair CYOA-style clinical cases with dynamic mapping tools to visualize user decision-making patterns and Evaluate their effectiveness as a pedagogical tool. We developed CYOA branched narrative case studies to simulate clinical decision-making in the management of high-dose methotrexate treatment for patients with pediatric leukemia. Following completion of the case study, user decision-making was mapped using R-based DiagrammeR software, which visualized the paths taken by each participant through the case studies. The maps effectively visualized decision-making at each stage of the CYOA cases, revealing a variety of user pathways and decision-making behaviors. Analysis showed that 25.9% of users achieved expert-level decision-making, whereas 51.9% made at least one incorrect decision that led to adverse outcomes. Furthermore, the maps enabled comparisons of behavior between different cohorts, highlighting how various interventions influenced decision patterns within the case study. Notably, these visualizations and data tables were generated in under ten seconds, providing rapid insights into user performance. This study demonstrates the potential of combining CYOA-style case studies with dynamic mapping tools to assess clinical reasoning in a more nuanced and interactive way.

**Supplementary Information:**

The online version contains supplementary material available at 10.1007/s40670-025-02505-6.

## Introduction

Developing a framework for clinical reasoning is a key tenet of medical training [1]. Courses and instructional programs are thus designed to guide trainees to integrate analytical skills, domain knowledge, basic sciences, and personal experiences when determining diagnoses and describing treatment plans [2, 3]. These curricula aim to help students advance their clinical reasoning skills from the novice approach of confirming pre-existing hypotheses via incomplete data analysis to the expert practice of using data to inform diagnostic hypotheses [3, 4].

While it is critical for medical training programs to aid students in developing robust clinical reasoning skills, evaluating trainees’ progress in this area is often challenging. Authentic clinical reasoning scenarios are often quite complex and thus difficult to easily evaluate at scale. Consequently, assessment of critical reasoning across large cohorts has often been achieved through multiple choice questions (MCQs; [5]) with perhaps the most well-known example being the United States Medical Licensing Examination (USMLE) board exams. MCQs provide psychometric stability and rapid evaluation [6, 7] but often fail to represent the constellation of choices available in real-life clinical scenarios. The limitations imposed by MCQs can be circumvented by using innovative and rigorous alternatives such as script concordance testing [8], extended matching questions [4, 9], and comprehensive integrative puzzles [10]. These methods more closely approximate the level of sophisticated reasoning required in a clinical encounter by presenting students with a patient vignette and an array of possible treatment options. Like MCQs, however, these approaches are not able to replicate the dynamic information gathering and responsiveness that occurs during the course of treatment [11]. Although these approaches have many options available, there is typically only one correct answer, which does not mirror the range of acceptable options applied during clinical problem solving [12, 13]. Additionally, the questions are static and siloed from one another, meaning that the input from one question does not affect the reasoning or logic of subsequent questions. This is a major divergence from real-world clinical reasoning, where downstream decisions are reliant on the ones made earlier.

A Choose Your Own Adventure (CYOA) format is an intriguing resolution to static forms of assessment. CYOA books allow readers to navigate a non-linear story through active decision-making, resulting in different scenarios and endings [14]. This format has been adapted for use in educational contexts as diagnostic branched tree (DBT) assessments [15, 16] or as CYOA-style patient case activities [17, 18]. During these assessments, students are presented with multiple treatment options that then branch to outcomes related to their choice. These assessments can be designed to test specific decision points or to evaluate how students respond in certain situations [19, 20].

Branching assessments have been used in several medical education contexts and have had a positive impact on both qualitative and quantitative measures. Psychiatry residents using DBT virtual patients improved their clinical understanding and viewed the learning experience as positive [21]. Similarly, pharmacy students have been evaluated using CYOA assessments for topics ranging from diabetes management [22] to insulin delivery [17] and hypertension [23], nursing students in cancer care [24] and empathetic communication [25], and podiatry students for x-ray management [26]. While each of these instances demonstrates that branching assessments are useful pedagogical tools that are growing in popularity, they are often challenging to create [16, 27] and evaluation of the critical decision points within the storyline [28] can be difficult given the various permutations resulting from each decision.

Here, we present a system that combines a CYOA case study with an automated map of the clinical reasoning behavior that users employed within the case. We first created simulated cases in a CYOA format based on clinical data and then applied our visualization system to the results. Management of a common yet complicated chemotherapy regimen, high-dose methotrexate, was chosen as a proof-of-principle because the stepwise nature of the decisions involved in this treatment was amenable to the CYOA format. We hypothesized that this system would be able to effectively display a range of clinical reasoning choices within a case as well as the different choices made by users while navigating a branched-style case study.

## Materials and Methods

### “Choose Your Own Adventure” Case Study Development

**Fig. 1:**
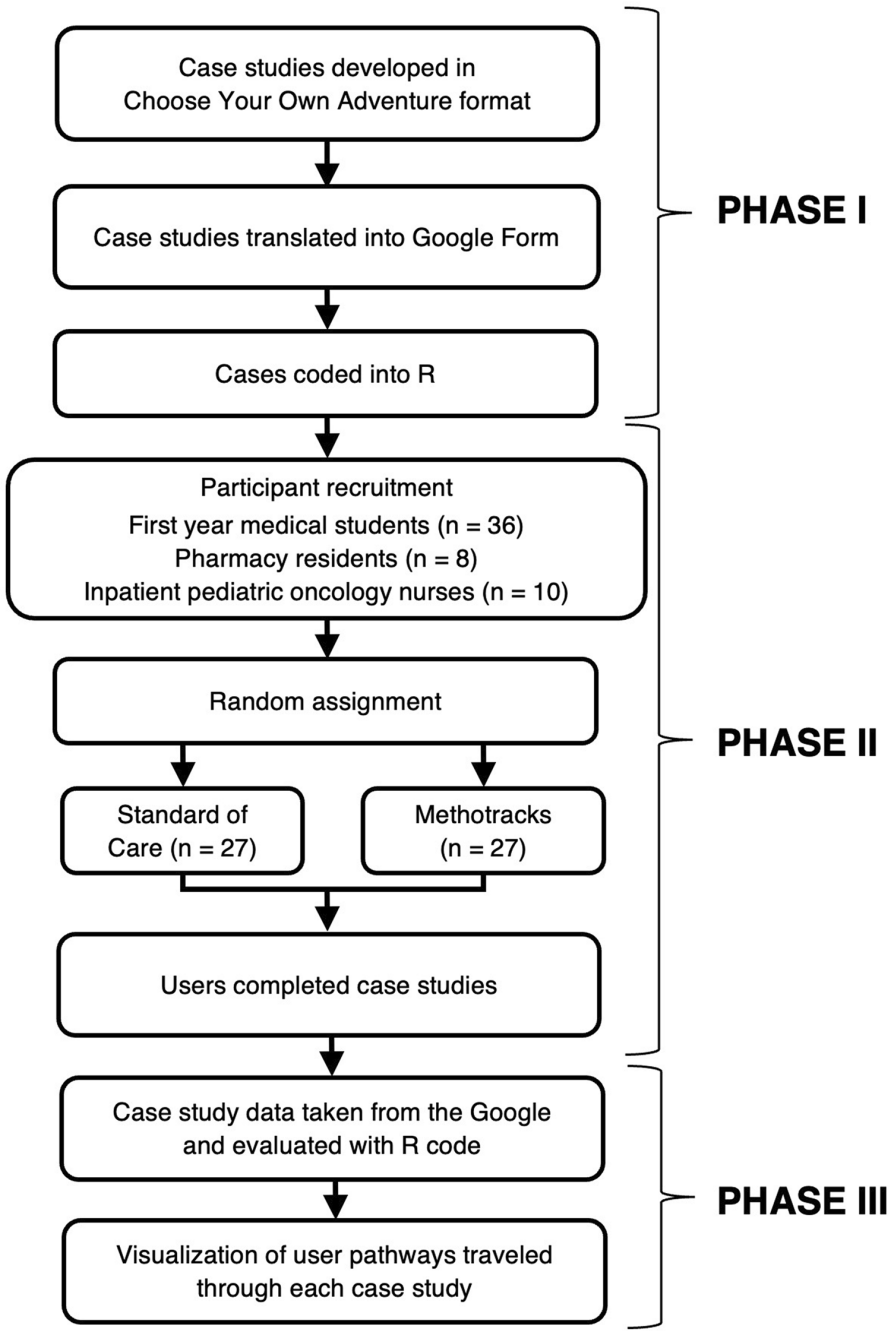
Flowchart of study design. Case studies about the delivery of high-dose methotrexate were developed as branched narrative multiple-choice questions (e.g., Choose Your Own Adventure format; Phase I). The text and logic of the cases were moved into a Google Form which routed users to different questions based on their input. Participants were recruited to complete each case using either a standard-of-care flowchart or Methotracks (Phase II). User’s answers were stored within a Google Sheet linked to the Form. Each case was mapped in R, and code was developed to evaluate the user data. Following completion of the study period, the case data were evaluated with R, and the routes through each case study were visualized (Phase III)

Virtual patient scenarios were previously developed to test the efficacy of a computer-based decision support tool for the delivery of high-dose methotrexate [29]. Four total scenarios were developed as Choose Your Own Adventure (CYOA) narrative multiple-choice quizzes, hereafter referred to as CYOA case studies (Fig. 1, Phase I). Only one scenario will be discussed in the context of this paper. The introduction to the case described a patient with acute lymphoblastic leukemia and included the patient’s initial laboratory data. The first prompt provided the methotrexate level, creatinine level, fluid intake, urine output, urine pH, and urine specific gravity at 24 h after the start of the high-dose methotrexate, the first clinical decision point. The first question within the prompt asked if hydration should be increased (Yes or No; Online Resource 1 Fig. 1A); the next question asked users to choose from a menu of distinct treatment options regarding the timing of when levels should next be Evaluated and if additional interventions were required. These two questions were repeated for each prompt, which corresponded to 6-h windows following the initial treatment. The menu of clinical options (8 minimum to 14 maximum; Online Resource 1 Table 1) changed based on the time point of the prompt. These options ranged from checking levels at various intervals (6, 12, 18, or 24 h) to administering different concentrations of leucovorin or discharging the patient. The routing for each treatment option is visible in the author view (Online Resource 1 Fig. 1B), illustrating the CYOA nature of the cases. Each treatment decision routes users to the corresponding outcome prompt. For example, choosing “Obtain the next level in 6 h” routes the user to Sect. 3, which corresponds to the 30-h timepoint. Each endpoint corresponded to different outcomes, such as waiting too long to check levels, discharging inappropriately, or successfully treating the patient. To simplify the case, some options route to the same endpoint. For instance, all choices to administer leucovorin at 100 mg/m^2^/dose lead to Sect. 10 because this dosage, administered too early, would harm the patient.

**Table 1:** User behavior quantification in a CYOA case. The numbers of users (*n*) and percentages of users (pct) demonstrating a specific type of decision-making behavior at each node or decision point in Scenario I. “Tolerable” indicates the users who selected expert and/or unharmful responses within each prompt. “Expert” indicates users who selected only the expert responses. “Incorrect” indicates users who made a choice within the case study that would lead to an adverse reaction in the patient

Node	Decision point	Decision-making behavior					
		Tolerable (*n*)	Tolerable (pct)	Expert (*n*)	Expert (pct)	Incorrect (*n*)	Incorrect (pct)
1	24Hr	54	100.0	54	100.0	0	0.0
3	24Hr fluid	54	100.0	53	98.1	0	0.0
10	42Hr	43	79.6	26	48.1	11	20.4
12	42Hr fluid	43	79.6	17	31.5	11	20.4
13	48Hr	41	75.9	14	25.9	13	24.1
15	48Hr fluid	41	75.9	14	25.9	13	24.1
16	54Hr	41	75.9	14	25.9	13	24.1
17	54Hr fluid	41	75.9	14	25.9	13	24.1
19	60Hr	33	61.1	14	25.9	21	38.9
21	60Hr fluid	33	61.1	14	25.9	21	38.9
FINISH	66Hr	26	48.1	14	25.9	28	51.9

The logic of the CYOA case was developed by assigning each prompt and each endpoint an identifier. These identifiers were used to “tag” each treatment option so that it would link to either another prompt or an end point. Using this information as a preliminary outline, each case was then converted into a Google Forms survey. The “Go to section based on answer” option was used to link treatment options to different prompts or endings. All endpoints display the same message to shield users from the diagnostic meaning, but the section number indicates which endpoint was selected. The full case is available in Online Resource 2. After survey submission, these answers were stored in a Google Spreadsheet.

### Assessment of the CYOA Cases

Each prompt with the CYOA case had one “expert” route, defined as the choices within the Case study made by three pediatric oncologists who specialize in the treatment of leukemia; there was 100% concordance for each prompt. Other choices within each prompt were tagged as “tolerable” choices, meaning that the choice was not aligned with the expert decision but would not cause immediate harm to the patient, or “incorrect” choices, meaning that the choice would cause harm to the patient.

### Creating CYOA Maps

**Fig. 2:**
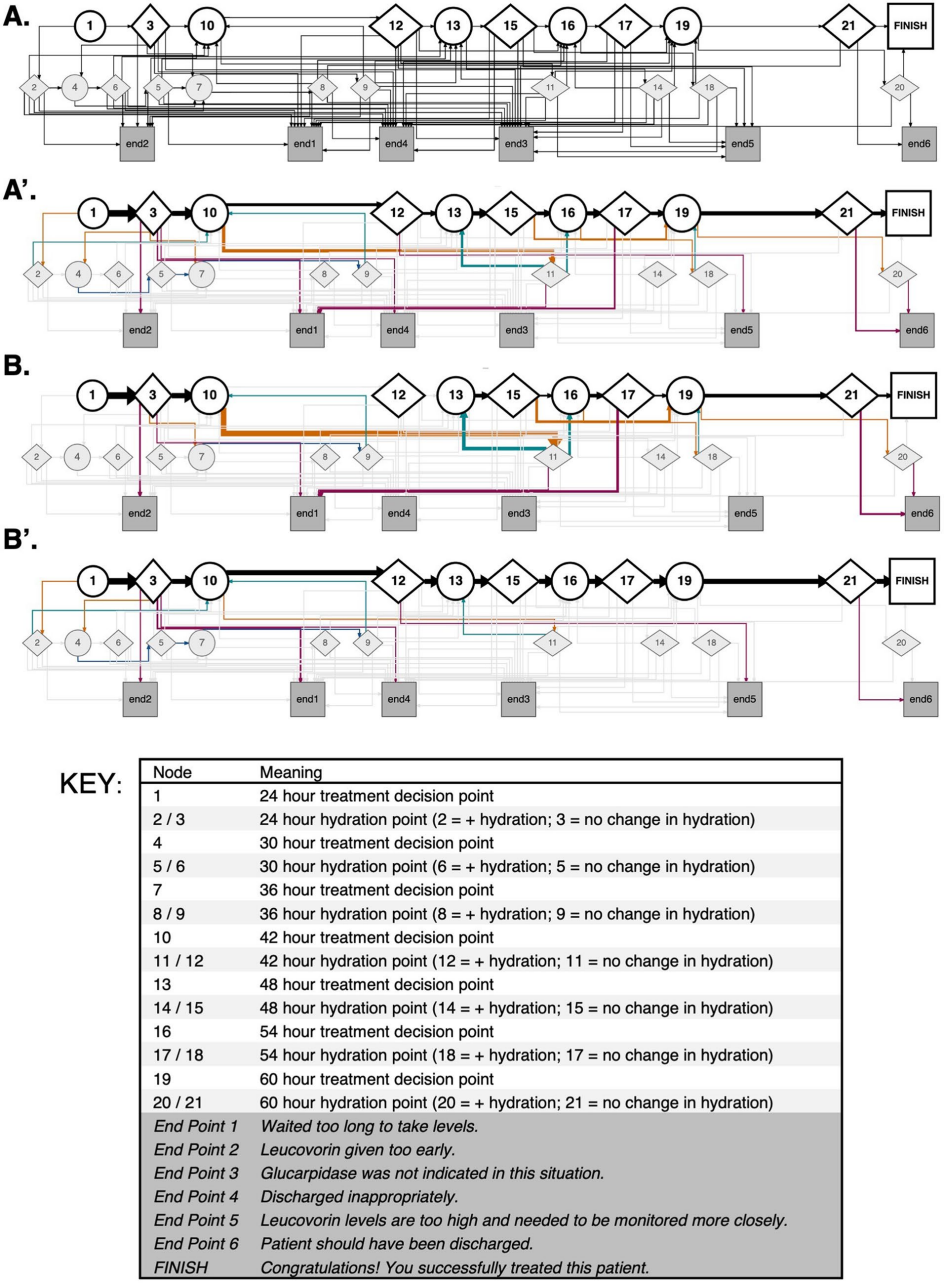
User behavior in a CYOA case is revealed through case mapping. **A** Base map of Scenario 1 displaying all nodes and possible routes. The starting time point (24 h) is represented by the 1 node; additional timepoints are represented by circles. Hydration choices (yes or no) are indicated by diamonds. Endings are indicated by dark gray squares. Possible routes between decision points are shown as lines. The expert path through the case study is indicated at the top of the image and ends with the FINISH square. **A’** Total user behavior in Scenario 1. Gray lines indicate paths not taken; black lines indicate users on the expert route; teal lines indicate users who made a decision that deviates from the expert path; magenta lines indicate users who made an incorrect decision. The thickness of the line corresponds to the proportion of users making the choice. **B** User (*n* = 27) behavior when completing the case using the SOC flowchart. **B’** User (*n* = 27) behavior when completing the case using the Methotracks app

The map of the CYOA case study was created by tagging each hydration prompt, treatment prompt, and ending point as a Node (Fig. 1, Phase I). The paths (i.e., Edges) in each scenario are defined by how nodes connect to one another. The Node and Edge data are combined to make a map using the R-based DiagrammeR package (Fig. 2A). Attributes of the map (such as colors, shape sizes, map direction, line angling, etc.) can be defined within the DiagrammeR package. Treatment nodes are indicated by circles, hydration nodes by diamonds, and end points as squares. The uppermost line of each map shows the expert-reasoning path (discussed further below); incorrect case end points are displayed in the bottom line of each map. The node that indicates the final correct treatment option within the case study is defined as the *FINISH* node. Paths that users travel are represented by black lines; untraveled paths are represented by gray lines. Path lines increase in weight based on the frequency of users who take the path.

### Participants

Participants for this study included first-year medical students (*n* = 36); pharmacy residents (*n* = 8); and inpatient pediatric oncology nurses (*n* = 10; Fig. 1, Phase II). The participant groups were chosen based on their unfamiliarity with methotrexate dosing decisions in an effort to decrease bias. The 54 total participants were randomized to either the standard of care (SOC) group (18 medical students; 4 pharmacy residents; 5 nurses), which received a Children’s Oncology Group high-dose methotrexate protocol AALL1732 flowchart [30] before completing the case study, or the Methotracks group (18 medical students; 4 pharmacy residents; 5 nurses), which received access to the Methotracks app [29] before completing the case study (Online Resource 1 Table 2). Participants were stratified by type, so that there were equal numbers of medical students, nurses, and pharmacy residents in each of the two groups. Users were assigned a random identification number that was entered at the start of each case study to ensure that the data was de-identified. This study was approved by the Johns Hopkins University Institutional Review Board, IRB00203619. Following completion of all four case studies, participants received a $20 Amazon gift card.

### Mapping the CYOA Cases

R code was used to retrieve the spreadsheet of user data for the scenario (Fig. 1, Phase III). Code was developed using *readxl* and *tidyr* packages to map user behavior in each case study onto the corresponding DiagrammeR map. Additional code was developed to generate tables and bar charts to assess user behavior. Beta testing to refine the code was done with volunteer users. The code is available at https://github.com/cahanlon/CYOA and a “How To” guide can be accessed at https://rpubs.com/cahanlon/CYOAHowToGuide.

## Results

### CYOA Map Visualization

Each map displays the entire range of possible user behavior within each CYOA Case study in less than 10 s. The treatment prompts of the case study are represented as a circular timepoint node (Fig. 2(A’); i.e., Node 1, Node 10, Node 13, etc.) and timepoint hydration choices are represented as diamonds (Fig. 2(A’); i.e. Node 2, Node 3, etc.). The meaning of each node is shown in the map key. Users move through the case study by making decisions at various points: after viewing the first patient description and treatment prompts (Node 1: 24-h treatment decision), they choose whether to increase or maintain hydration (Node 2: increase, Node 3: no change). These choices are represented by lines emanating from the nodes with options leading to subsequent treatment timepoints or endpoints. For instance, there are seven total lines emanating from Node 3; routes to Node 4, Node 7, or Node 10 reflect different decision points, while routes leading to End1–End4 represent the case’s possible conclusions. Because several choices may lead to the same option, some CYOA routes collapse into the same endpoint (Online Resource 1 Fig. 1B).

The maps were designed to showcase decision-making patterns. The top row of the CYOA map shows the choices that experts (pediatric oncologists) make: no change in hydration at 24 h, treatment at 42 h, increase hydration at 42 h, treatment at 48 h, and so on (Fig. 2(A)). Tolerable decisions (those that do not cause immediate harm to the patient) are represented by nodes in the middle row of the map. Incorrect decisions (those that would cause harm to the patient) are represented as endpoint nodes in the bottom row of the map.

### User Behavior Within CYOA Case Studies

Following completion of the CYOA cases, the behavior of 54 users was superimposed on the map of the scenario (Fig. 2(A’)), revealing a range of decision-making paths indicated by varying line colors and thicknesses. For example, most users opted correctly not to increase hydration (thick black line from Node 1 to Node 3), while fewer chose to increase it (thin orange line from Node 1 to Node 2). Deviations from the expert path (black lines) indicate tolerable (orange lines) or incorrect decisions (magenta lines). Interestingly, many users returned to the expert decision-making path, which is observed in the teal lines returning to Nodes 10 and 13. Altogether, the CYOA map can depict different journeys and outcomes of users participating in a CYOA case study.

While the map is useful to understand user behavior through the Case study at a glance, the same code simultaneously generates data tables with more detailed information. Within the case study, expert decision-making was demonstrated by 25.9% of users (Table 1). In other words, 25.9% of users exclusively visited the nodes on the top row of the map (Fig. 2(A’)). Tolerable decision-making was exercised by 48.1% of users (Table 1), reflecting those who initially erred but returned to the expert path. This behavior is also visible in the map in the orange line leading to the Node 11 hydration point and the teal line leading back to the Node 13 treatment point (Fig. 2(A’)). Incorrect decision-making was used by 51.9% of users (Table 1), meaning they chose an ending that would be detrimental to the patient. Other descriptive information can also be generated, such as the number of nodes visited (Online Resource 1 Table 3), the distribution of endings selected by users (Online Resource 1 Table 4), and the frequency of each route taken by users (Online Resource 1 Table 5).

### Comparing User Behavior Between Groups

A powerful feature of this system is that it can quickly generate visual comparisons between different types of users. In this example, we were testing if users traversed the CYOA case study differently when using a standard-of-care (SOC) flowchart versus Methotracks (MTX) a computer-based decision support tool [29]. The map revealed distinct patterns between the groups (Fig. 2(B-B’); Online Resource 1 Tables 2–4). For example, at the 42-h decision point (Node 10), all SOC users deviated from the expert path (orange line to Node 11), while most MTX users remained on it (black line to node 12). Additionally, more SOC users routed to End Point 1, indicating delayed patient evaluation. While similar trends have been noted in previous studies [29], the map captures the dynamic decision-making process and shows how each cohort navigated the case. The data can also be disaggregated by demographic group (nurses, pharmacology residents, and medical students (Online Resource 1 Table 3 and Online Resource 1 Fig. 2). Notably, more MTX users remained on the expert path than the SOC users, regardless of level of training (Online Resource 1 Fig. 2).

## Discussion

Diagnostic branched tree assessment is a method used to evaluate a learner’s clinical reasoning by guiding them through a series of branching decisions that simulate the diagnostic process. Each decision point represents a choice based on patient information, where the learner must select the most appropriate next step. This approach is useful because it mirrors real-world diagnostic decision-making and can reveal not only the final diagnosis but also the reasoning process behind it. Choose Your Own Adventure (CYOA)-style assessments build upon DBT by increasing the number of options at each decision point, offering a more complex and nuanced exploration of clinical reasoning. Learners enjoy the CYOA assessments, and participation in this type of activity increases academic performance and engagement [31].

In this study, we developed a tool that takes data from CYOA case studies to generate maps that reflect clinical reasoning. These maps clearly illustrate the range of possible pathways within each case study and reveal patterns in decision-making. Trends, such as the proportion of users taking a particular path or the nodes where users diverge from the expert path, were easily identifiable. These data visualizations also provide a straightforward approach to compare two different approaches to a clinical problem. Traditionally, this type of data is presented in cumbersome tables, but our tool provides assessors with an intuitive, scalable graphic representation of user behavior. Data visualizations have been increasingly used across medical education and practice to facilitate understanding and improve outcomes. Mapping tools have been designed to identify gaps and redundancies within courses [32, 33] and to serve as dashboards for student learning [34]. The shared goal for these tools is to quickly disseminate information, reduce errors, streamline data interpretation, and improve outcomes, whether for students or patients. By tracing user behavior, the CYOA mapping tool revealed the structure and flow of clinical decision-making, supporting its value as a dynamic data visualization instrument.

To our knowledge, this study is the first to incorporate data visualizations with CYOA assessments to reveal decision-making patterns in complex clinical case studies. While CYOA assessments have been used in several medical education contexts [17, 22–26], this is the only study that has incorporated a data visualization of the CYOA map and user behavior. The integration of visual tools into CYOA-style assessments offers a powerful way to streamline the evaluation of complex decision-making processes and foster deeper clinical reasoning skills. The strength of this study lies in its ability to display numerous potential treatment pathways, including routes that diverge from and rejoin the expert path. This mimics the choice architecture inherent in clinical decision-making and supports the view that there is rarely only one “right” way to diagnose or treat a patient [11]. This approach contrasts with traditional assessments like multiple-choice questions, which tend to favor a single correct answer [5]. In clinical practice, physicians must tolerate uncertainty [35], and assessments promoting only one correct answer may inadvertently discourage flexibility. By incorporating CYOA assessments as formative tools with instructor coaching [2, 36], students can be exposed to a range of acceptable decision-making pathways and develop a tolerance for ambiguity, a critical skill in clinical practice [37, 38].

Building on this idea, our approach offers intriguing opportunities for both developing and assessing learning outcomes going forward, especially as it pertains to case-based learning (CBL). CBL discussions are typically led by a clinical instructor who prompts students to engage in clinical reasoning. Work by Gartmeier et al. [39] showed that most questions posed by clinical instructors in case-based learning scenarios are initial, rather than follow-up, and close-ended rather than open-ended. This creates a challenge for ensuring that CBLs encourage the development of deep clinical reasoning. Follow-up questions push students to view decision-making as an evolving process rather than a means to reach a single pre-defined conclusion. We envision this system being used in future applications as a formative assessment tool that promotes discussion of open-ended, reasoning-based questions. For example, while this pilot study mapped cohort-level progress through case studies, future iterations could be used in active-learning classrooms, where students complete a case study at the beginning of class and then analyze decision-making pathways, reflect on clinical reasoning strategies, and link the study back to the learning objectives of the unit. Using the map of user behavior, instructors could give targeted feedback on common decision-making pitfalls and reinforcement of expert reasoning strategies. This method could also easily be adapted for use in a flipped classroom: learners could complete the CYOA case study outside of class, and in-class sessions would be centered around the analysis of the CYOA map. Overall, the tool could help correct misconceptions, build new knowledge, and foster critical thinking in many medical education settings.

Future research will focus on how this tool can be operationalized in the classroom, exploring both learner and instructor attitudes toward its use. Another avenue for future exploration is applying this methodology to other domains that require dynamic and longitudinal patient management. Further refinement of the system will aim to determine the most effective ways to display decision-making maps for rapid evaluation [40] and optimize the number of options and branches within each decision point [27].

There were several limitations to this study. One major limitation was the small sample size and restriction to a single university, which may have influenced the pathways chosen by users. These choices could reflect a lack of familiarity with Methotracks or therapeutic management rather than true decision-making behaviors. Furthermore, the CYOA case study used in this study was administered through Google Forms which has a “go back” button that cannot be disabled. For this reason, we opted to blind users from the outcome of their treatment decision. While this decision gave us a more honest look at how users were traversing the case study, it limited our ability to gather insights into how users reflected on their decision-making process. Future iterations of this study will incorporate these changes to better understand how this system could be used in a classroom environment. A final limitation is the time-intensive nature of creating DBT and CYOA case studies, which require authors to account for multiple pathways and endings. The creation of the accompanying maps also requires proficiency in R-based coding.

Despite these challenges, the potential for DBT and CYOA assessments to more closely mimic real-world decision-making is high [22].Therefore, the issue becomes how to make these types of cases easier to create. One intriguing possibility is to pair case CYOA study development with artificial intelligence (AI), which could automate the creation of complex case scenarios and map interactions in real-time. AI could also be used with natural language processing to evaluate open-ended responses to CYOA prompts and route users to the appropriate scenario outcome. Such an AI-enhanced CYOA environment would offer a more realistic simulation of clinical practice, requiring trainees to formulate their own responses to open-ended prompts. AI is already being used in medical education to create virtual patients for diagnostic skills practice (see Dartmouth AI Patient Actor App [41]), generate exam-style questions [42], and serve as a tutor for problem-based learning [43]. Leveraging AI to develop CYOA-style cases could streamline the creation process and provide users with immediate feedback on their clinical reasoning. This integration of AI would ease the entry point for DBT and CYOA implementation while enhancing the quality and scope of data generated from these assessments. As medical education continues to embrace digital and AI-driven tools, the potential for CYOA and DBT assessments to transform clinical reasoning training is immense.

## Conclusions

This study demonstrates the feasibility of using a Choose Your Own Adventure style diagnostic branched tree assessment system to visualize and evaluate clinical reasoning processes. By integrating data visualizations with decision-making pathways, we provide a novel approach for assessing how users navigate complex clinical scenarios, offering a more dynamic alternative to traditional assessments. The system can help instructors identify critical decision points and foster deeper clinical reasoning through targeted feedback. Future research will explore broader applications of this tool in medical education and its potential to improve real-time assessment of clinical reasoning skills across different domains.

## Supplementary Information

Below is the link to the electronic supplementary material.

MOESM 1 Online Resource 1 (PDF 753 KB)

MOESM 2 Online Resource 2 (PDF 387 KB)

## Data Availability

The dataset and coding files for this data is available at https://osf.io/7vs36/?view_only=e6337ba2b47e4a15b7d485e58dbc3310.

## ABBREVIATIONS

AI: Artificial intelligence
DBT: Diagnostic branched tree
CYOA: Choose your own adventure
MTX: Methotracks intervention
SOC: Standard of care intervention
