# Supplementary material for: Visualizing Multi-Step Decision-Making at a Glance: Pairing Choose Your Own Adventure Style Simulated Cases with a Novel Mapping Framework
Source: Med Sci Educ. 2025 Sep 12;35(6):2985–94. doi: 10.1007/s40670-025-02505-6 (PMC12961062; doi:10.1007/s40670-025-02505-6)

Supplemental Table 1: Number of treatment options and endings for CYOA Cases 1-4

NODES		TREATMENT OPTIONS			
**Hour**	**Question**	**Case1**	**Case2**	**Case3**	**Case4**
24	Q1	14	11	14	11
30	Q2	14	11	11	11
36	Q3	14	11	11	11
42	Q4	14	11	8	*na*
48	Q5	14	11	11	*na*
54	Q6	14	11	9	*na*
60	Q7	14	11	8	*na*
66	Q8	*na*	11	*na*	*na*
	Endings	7	7	7	7

A.

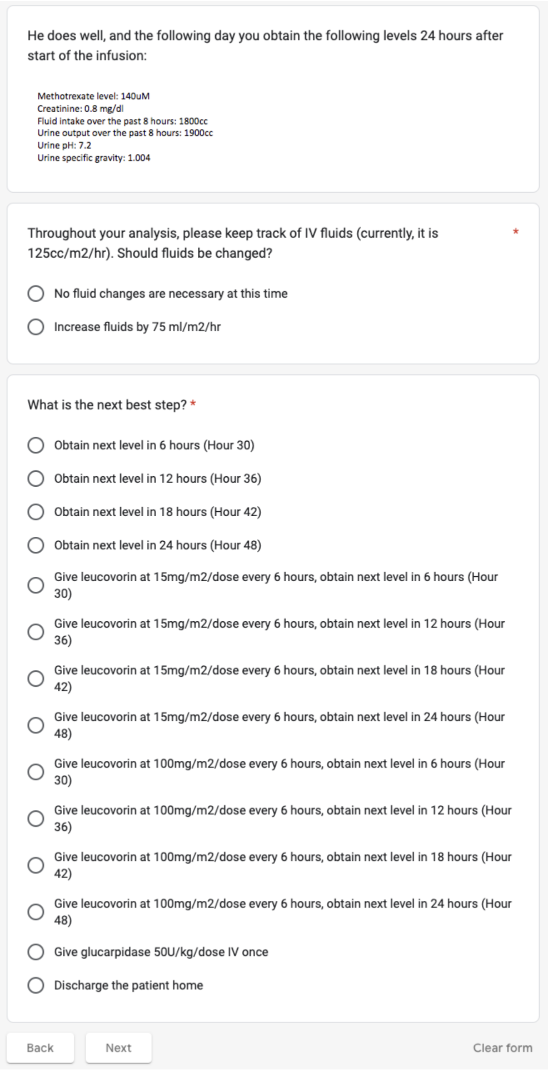
He does well, and the following day you obtain the following levels 24 hours after start of the infusion:

Methotrexate level: 140uM
Creatinine: 0.8 mg/dl
Fluid intake over the past 8 hours: 1800cc
Urine output over the past 8 hours: 1900cc
Urine pH: 7.2
Urine specific gravity: 1.004

Throughout your analysis, please keep track of IV fluids (currently, it is 125cc/m2/hr). Should fluids be changed? *

- No fluid changes are necessary at this time
- Increase fluids by 75 ml/m2/hr

What is the next best step? *

- Obtain next level in 6 hours (Hour 30)
- Obtain next level in 12 hours (Hour 36)
- Obtain next level in 18 hours (Hour 42)
- Obtain next level in 24 hours (Hour 48)
- Give leucovorin at 15mg/m2/dose every 6 hours, obtain next level in 6 hours (Hour 30)
- Give leucovorin at 15mg/m2/dose every 6 hours, obtain next level in 12 hours (Hour 36)
- Give leucovorin at 15mg/m2/dose every 6 hours, obtain next level in 18 hours (Hour 42)
- Give leucovorin at 15mg/m2/dose every 6 hours, obtain next level in 24 hours (Hour 48)
- Give leucovorin at 100mg/m2/dose every 6 hours, obtain next level in 6 hours (Hour 30)
- Give leucovorin at 100mg/m2/dose every 6 hours, obtain next level in 12 hours (Hour 36)
- Give leucovorin at 100mg/m2/dose every 6 hours, obtain next level in 18 hours (Hour 42)
- Give leucovorin at 100mg/m2/dose every 6 hours, obtain next level in 24 hours (Hour 48)
- Give glucarpidase 50U/kg/dose IV once
- Discharge the patient home

Back Next Clear form

B.

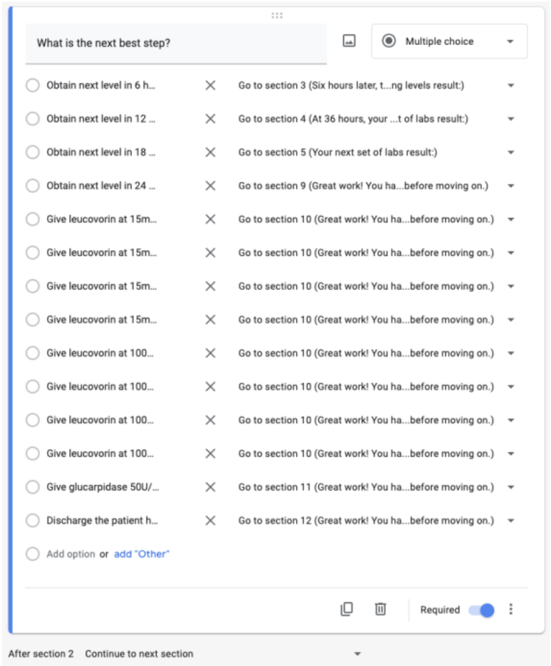
What is the next best step? Multiple choice

Obtain next level in 6 h...	Go to section 3 (Six hours later, t...ng levels result:)
Obtain next level in 12 ...	Go to section 4 (At 36 hours, your ...t of labs result:)
Obtain next level in 18 ...	Go to section 5 (Your next set of labs result:)
Obtain next level in 24 ...	Go to section 9 (Great work! You ha...before moving on.)
Give leucovorin at 15m...	Go to section 10 (Great work! You ha...before moving on.)
Give leucovorin at 15m...	Go to section 10 (Great work! You ha...before moving on.)
Give leucovorin at 15m...	Go to section 10 (Great work! You ha...before moving on.)
Give leucovorin at 15m...	Go to section 10 (Great work! You ha...before moving on.)
Give leucovorin at 100...	Go to section 10 (Great work! You ha...before moving on.)
Give leucovorin at 100...	Go to section 10 (Great work! You ha...before moving on.)
Give leucovorin at 100...	Go to section 10 (Great work! You ha...before moving on.)
Give leucovorin at 100...	Go to section 10 (Great work! You ha...before moving on.)
Give glucarpidase 50U/...	Go to section 11 (Great work! You ha...before moving on.)
Discharge the patient h...	Go to section 12 (Great work! You ha...before moving on.)

Add option or add "Other"

Required

After section 2 Continue to next section

Supplementary Figure 1: Screenshot of CYOA case study. A. User view of Case 1. Users were given patient data and presented with questions regarding fluid maintenance and methotrexate management. **B.** Author view of the same methotrexate management prompt.

Supplementary Table 2: User demographic by training (nurse, pharmacy resident, or medical student) and by treatment (SOC or MTX).

	n	pct.group	pct.total
Nurse.SOC	5	50	9.3
Nurse.MTX	5	50	9.3
PharmRes.SOC	4	50	7.4
PharmRes.MTX	4	50	7.4
MedStudent.SOC	18	50	33.3
MedStudent.MTX	18	50	33.3
SUM	54		

Supplementary Table 3: Number of nodes visited by users in CYOA Case 1. The average number of choices taken per prompt, the average number of nodes visited, and the number of unique nodes visited were calculated for each group of users. The range indicates the minimum and maximum number of choices taken or nodes visited.

USER	AVERAGE NUMBER OF NODES VISITED *(range)*	UNIQUE NODES VISITED	UNIQUE ENDS VISITED
TOTAL	8.4 *(3-15)*	24	6
SOC	7.6 *(3-13)*	18	4
MTX	9.1 *(3-15)*	22	6

Supplementary Table 4: Frequency of end prompts visited by users in CYOA Case 1.

End	Meaning	TOTAL (n)	TOTAL (%)	SOC (n)	SOC (%)	MTX (n)	MTX (%)
1	Waited too long for levels.	14	25.9	10	37.0	4	14.8
2	Leucovorin given too early.	5	9.3	4	14.8	1	3.7
3	Glucarpidase was not indicated in this situation.	0	0.0	0	0.0	0	0.0
4	Discharged inappropriately.	1	1.9	0	0.0	1	3.7
5	Leucovorin levels are too high and needed to be monitored more closely.	1	1.9	0	0.0	1	3.7
6	Patient should have been discharged.	7	13.0	6	22.2	1	3.7
7	Congratulations! You successfully treated this patient.	26	48.1	7	25.9	19	70.4

Supplementary Table 5: Frequency of end prompts visited by users in CYOA Case 1. Pathways are listed in the first column. Values indicate the number of users who took each pathway. Double asterisk (**) indicates the expert decision-making path.

Pathway	Frequency TOTAL	Frequency SOC	Frequency MTX
**START, No (24), 42Hr, Yes (42), 48Hr, No (48), 54Hr, No (54), 60Hr, No (60), FINAL	18	0	18
START, No (24), 42Hr, No (42), 54Hr, No (54), End 1	7	7	0
START, No (24), 42Hr, No (42), 48Hr, No (48), 60Hr, No (60), FINAL	6	6	0
START, No (24), End 1	5	1	4
START, No (24), End 2	5	4	1
START, No (24), 42Hr, No (42), 48Hr, No (48), 54Hr, No (54), 60Hr, No (60), End 6	2	2	0
START, No (24), End 4	1	0	1
START, No (24), 30Hr, No (30), 36Hr, No (36), 42Hr, Yes (42), 48Hr, No (48), 54Hr, No (54), 60Hr, No (60), End 6	1	0	1
START, No (24), 36Hr, No (36), 42Hr, No (42), 48Hr, No (48), 54Hr, No (54), 60Hr, No (60), End 6	1	1	0
START, No (24), 42Hr, No (42), 48Hr, No (48), 54Hr, No (54), End 1	1	1	0
START, No (24), 42Hr, No (42), 48Hr, No (48), 54Hr, Yes (54), 60Hr, No (60), End 6	1	1	0
START, No (24), 42Hr, No (42), 48Hr, No (48), 60Hr, No (60), End 6	1	1	0
START, No (24), 42Hr, No (42), 48Hr, No (48), 60Hr, Yes (60), End 6	1	1	0
START, No (24), 42Hr, No (42), 54Hr, No (54), 60Hr, No (60), FINAL	1	1	0
START, No (24), 42Hr, No (42), End 1	1	1	0
START, No (24), 42Hr, Yes (42), End 5	1	0	1
START, Yes (24), 42Hr, No (42), 48Hr, No (48), 54Hr, No (54), 60Hr, No (60), FINAL	1	0	1

	Point	n.Tol. SOC	pct.Tol. SOC	n.Tol. MTX	pct.Tol. MTX	n.Expert Associated. SOC	pct.Expert Associated. SOC	n.Expert Associated. MTX	pct.Expert Associated. MTX	n.Expert. SOC	pct.Expert. SOC	n.Expert. MTX	pct.Expert. MTX	n.Incorrect. SOC	pct. Incorrect. SOC	n.Incorrect. MTX	pct.Incorrect. MTX
NURSES (n = 10)	Hr24	5	100.0	5	100.0	5	100.0	5	100.0	5	100.0	5	100.0	0	0.0	0	0.0
	Hr24 fluid	5	100.0	5	100.0	5	100.0	5	100.0	5	100.0	5	100.0	0	0.0	0	0.0
	Hr42	2	40.0	4	80.0	2	40.0	4	80.0	1	20.0	4	80.0	3	60.0	1	20.0
	Hr42 fluid	2	40.0	4	80.0	0	0.0	4	80.0	0	0.0	4	80.0	3	60.0	1	20.0
	Hr48	2	40.0	4	80.0	1	20.0	4	80.0	0	0.0	3	60.0	3	60.0	1	20.0
	Hr48 fluid	2	40.0	4	80.0	1	20.0	4	80.0	0	0.0	3	60.0	3	60.0	1	20.0
	Hr54	2	40.0	4	80.0	1	20.0	4	80.0	0	0.0	3	60.0	3	60.0	1	20.0
	Hr54 fluid	2	40.0	4	80.0	1	20.0	4	80.0	0	0.0	3	60.0	3	60.0	1	20.0
	Hr60	1	20.0	4	80.0	1	20.0	4	80.0	0	0.0	3	60.0	4	80.0	1	20.0
	Hr60 fluid	1	20.0	4	80.0	1	20.0	4	80.0	0	0.0	3	60.0	4	80.0	1	20.0
	End7	1	20.0	4	80.0	1	20.0	4	80.0	0	0.0	3	60.0	4	80.0	1	20.0
	AVE	***2.3***	***45.5***	***4.2***	***83.6***	***1.7***	***34.5***	***4.2***	***83.6***	***1.0***	***20.0***	***3.5***	***70.9***	***2.7***	***54.5***	***0.8***	***16.4***
PHARM (n = 8)	Hr24	4	100.0	4	100.0	4	100.0	4	100.0	4	100.0	4	100.0	0	0.0	0	0.0
	Hr24 fluid	4	100.0	4	100.0	4	100.0	4	100.0	4	100.0	4	100.0	0	0.0	0	0.0
	Hr42	4	100.0	4	100.0	4	100.0	4	100.0	1	25.0	4	100.0	0	0.0	0	0.0
	Hr42 fluid	4	100.0	4	100.0	0	0.0	4	100.0	0	0.0	4	100.0	0	0.0	0	0.0
	Hr48	2	50.0	4	100.0	1	25.0	4	100.0	0	0.0	3	75.0	2	50.0	0	0.0
	Hr48 fluid	2	50.0	4	100.0	1	25.0	4	100.0	0	0.0	3	75.0	2	50.0	0	0.0
	Hr54	2	50.0	4	100.0	2	50.0	4	100.0	0	0.0	3	75.0	2	50.0	0	0.0
	Hr54 fluid	2	50.0	4	100.0	2	50.0	4	100.0	0	0.0	3	75.0	2	50.0	0	0.0
	Hr60	2	50.0	4	100.0	2	50.0	4	100.0	0	0.0	3	75.0	2	50.0	0	0.0
	Hr60 fluid	2	50.0	4	100.0	2	50.0	4	100.0	0	0.0	3	75.0	2	50.0	0	0.0
	End7	2	50.0	4	100.0	2	50.0	4	100.0	0	0.0	3	75.0	2	50.0	0	0.0
	AVE	***2.7***	***68.2***	***4.0***	***100.0***	***2.2***	***54.5***	***4.0***	***100.0***	***0.8***	***20.5***	***3.4***	***84.1***	***1.3***	***31.8***	***0.0***	***0.0***
MED (n = 36)	Hr24	18	100.0	18	100.0	18	100.0	18	100.0	18	100.0	18	100.0	0	0.0	0	0.0
	Hr24 fluid	18	100.0	18	100.0	18	100.0	17	94.4	18	100.0	17	94.4	0	0.0	0	0.0
	Hr42	16	88.9	13	72.2	16	88.9	13	72.2	7	38.9	9	50.0	2	11.1	5	27.8
	Hr42 fluid	16	88.9	13	72.2	0	0.0	12	66.7	0	0.0	9	50.0	2	11.1	5	27.8
	Hr48	16	88.9	12	66.7	11	61.1	12	66.7	0	0.0	8	44.4	2	11.1	6	33.3
	Hr48 fluid	16	88.9	12	66.7	11	61.1	12	66.7	0	0.0	8	44.4	2	11.1	6	33.3
	Hr54	16	88.9	12	66.7	10	55.6	12	66.7	0	0.0	8	44.4	2	11.1	6	33.3
	Hr54 fluid	16	88.9	12	66.7	9	50.0	12	66.7	0	0.0	8	44.4	2	11.1	6	33.3
	Hr60	10	55.6	12	66.7	10	55.6	12	66.7	0	0.0	8	44.4	8	44.4	6	33.3
	Hr60 fluid	10	55.6	12	66.7	9	50.0	12	66.7	0	0.0	8	44.4	8	44.4	6	33.3
	End7	4	22.2	11	61.1	4	22.2	11	61.1	0	0.0	8	44.4	14	77.8	7	38.9
	AVE	***14.2***	***78.8***	***13.2***	***73.2***	***10.5***	***58.6***	***13.0***	***72.2***	***3.9***	***21.7***	***9.9***	***55.1***	***3.8***	***21.2***	***4.8***	***26.8***

Supplementary Table 6: The numbers of users (n) and percentages of users (%) demonstrating a specific type of decision-making behavior at each node or decision point in Scenario I by demographic type. “Tolerable” indicates the users who selected expert and/or unharmful responses within each prompt. “Expert Associated” indicates the users on the expert path at a given node. “Expert” indicates users who selected only the expert responses. “Incorrect” indicates users who made a choice within the case study that would lead to an adverse reaction in the patient. Users are broken down by training (nurse, pharmacy resident, or medical student) and by treatment (SOC or MTX).

Supplementary Figure 2: Scenario I expert users by demographic. Users are broken down by training (nurse, pharmacy resident, or medical student) and by treatment (SOC or MTX). The plot indicates the percent of users at each time point who remained on the expert path (users who selected only the expert responses), equivalent to the top line of the CYOA map.

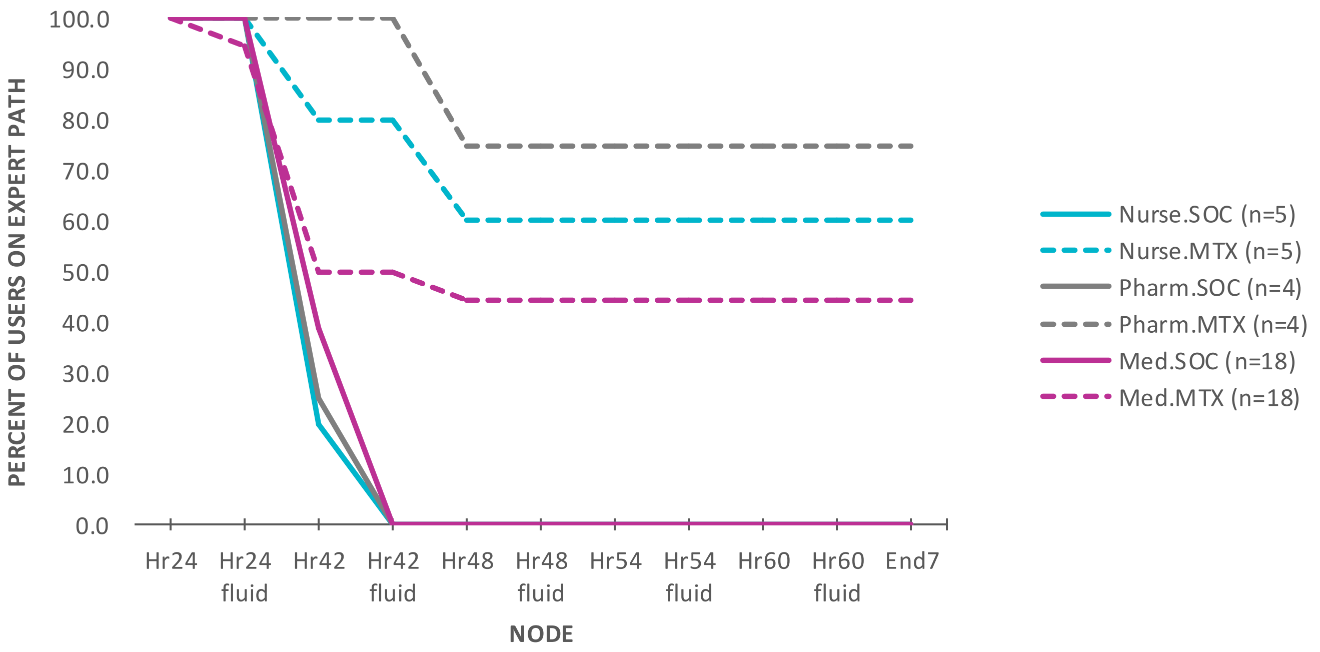

Supplement: Supplementary file 1 — Online Resource 1 (PDF 753 KB) [file 40670_2025_2505_MOESM1_ESM.pdf]
