# Supplementary material for: Visualizing Multi-Step Decision-Making at a Glance: Pairing Choose Your Own Adventure Style Simulated Cases with a Novel Mapping Framework
Source: Med Sci Educ. 2025 Sep 12;35(6):2985–94. doi: 10.1007/s40670-025-02505-6 (PMC12961062; doi:10.1007/s40670-025-02505-6)
Supplement: Supplementary file 2 — Online Resource 2 (PDF 387 KB) [file 40670_2025_2505_MOESM2_ESM.pdf]

MTX Scenario I

* Indicates required question

1. Please enter your unique identifier below: *

An 18 year old boy with high risk pre-B ALL is admitted for his first dose of high dose methotrexate, and is started on IV fluids at 125cc/m2/hour, with urinalysis performed with every void. Prior to starting the infusion, his nurse comes to you with the results of his urinalysis and baseline serum creatinine:

CHEM PROFILE		
Creatinine	Latest Range: 0.6-1.3 mg/dL	0.7

URINALYSIS	
Appearance, Urine	Clear
Bacteria, Urine	None
Bilirubin, Urine, ...	Negative
Color, Urine	Yellow
Crystals, Urine	None
Epithelial Cells, ...	0 *
Glucose, Urine, Se...	Negative
Granular Casts, Urine	0
Hemoglobin Pigment...	Negative
Hyaline Casts, Urine	0
Ketones, Urine, Qu...	Negative
Leukocyte Esterase...	Negative
Mucous Threads, Urine	
Nitrite, Urine	Negative
Protein Semiquant, UA	Negative
Red Blood Cells, UA	0
Red Blood Cells, U...	0
Specific Gravity, ...	1.009
Urine pH	7.0
Urobilinogen, Urine	<=1.0
White Blood Cells, UA	0
White Blood Cells,...	0

He does well, and the following day you obtain the following levels 24 hours after start of the infusion:

Methotrexate level: 140uM
Creatinine: 0.8 mg/dl
Fluid intake over the past 8 hours: 1800cc
Urine output over the past 8 hours: 1900cc
Urine pH: 7.2
Urine specific gravity: 1.004

2. Throughout your analysis, please keep track of IV fluids (currently, it is 125cc/m2/hr). Should fluids be changed? *

Mark only one oval.

- No fluid changes are necessary at this time
- Increase fluids by 75 ml/m2/hr

3. What is the next best step? *

Mark only one oval.

- Obtain next level in 6 hours (Hour 30) *Skip to question 4*
- Obtain next level in 12 hours (Hour 36) *Skip to question 7*
- Obtain next level in 18 hours (Hour 42) *Skip to question 10*
- Obtain next level in 24 hours (Hour 48)
  Skip to section 9 (Great work! You have now reached the end of Scenario I. Please ensure that you hit "submit" before moving on.)
- Give leucovorin at 15mg/m2/dose every 6 hours, obtain next level in 6 hours (Hour 30)
  Skip to section 10 (Great work! You have now reached the end of Scenario I. Please ensure that you hit "submit" before moving on.)
- Give leucovorin at 15mg/m2/dose every 6 hours, obtain next level in 12 hours (Hour 36)
  Skip to section 10 (Great work! You have now reached the end of Scenario I. Please ensure that you hit "submit" before moving on.)
- Give leucovorin at 15mg/m2/dose every 6 hours, obtain next level in 18 hours (Hour 42)
  Skip to section 10 (Great work! You have now reached the end of Scenario I. Please ensure that you hit "submit" before moving on.)
- Give leucovorin at 15mg/m2/dose every 6 hours, obtain next level in 24 hours (Hour 48)
  Skip to section 10 (Great work! You have now reached the end of Scenario I. Please ensure that you hit "submit" before moving on.)
- Give leucovorin at 100mg/m2/dose every 6 hours, obtain next level in 6 hours (Hour 30)
  Skip to section 10 (Great work! You have now reached the end of Scenario I. Please ensure that you hit "submit" before moving on.)
- Give leucovorin at 100mg/m2/dose every 6 hours, obtain next level in 12 hours (Hour 36)
  Skip to section 10 (Great work! You have now reached the end of Scenario I. Please ensure that you hit "submit" before moving on.)
- Give leucovorin at 100mg/m2/dose every 6 hours, obtain next level in 18 hours (Hour 42)
  Skip to section 10 (Great work! You have now reached the end of Scenario I. Please ensure that you hit "submit" before moving on.)
- Give leucovorin at 100mg/m2/dose every 6 hours, obtain next level in 24 hours (Hour 48)
  Skip to section 10 (Great work! You have now reached the end of Scenario I. Please ensure that you hit "submit" before moving on.)
- Give glucarpidase 50U/kg/dose IV once
  Skip to section 11 (Great work! You have now reached the end of Scenario I. Please ensure that you hit "submit" before moving on.)
- Discharge the patient home
  Skip to section 12 (Great work! You have now reached the end of Scenario I. Please ensure that you hit "submit" before moving on.)

Six hours later, the following levels result:

30 hour methotrexate level: 80uM
Creatinine: 0.7mg/dl
Fluid intake over the past 8 hours: 2000cc
Urine output over the past 8 hours: 1900cc
Urine pH: 7.3
Urine specific gravity: 1.008

4. Should fluids be changed? *

Mark only one oval.

- No fluid changes are necessary at this time
- Increase fluids by 75 ml/m2/hr

5. What is the next best step? *

Mark only one oval.

- Obtain next level in 6 hours (Hour 36) *Skip to question 7*
- Obtain next level in 12 hours (Hour 42) *Skip to question 10*
- Obtain next level in 18 hours (Hour 48) *Skip to question 13*
- Obtain next level in 24 hours (Hour 54)
  Skip to section 9 (Great work! You have now reached the end of Scenario I. Please ensure that you hit "submit" before moving on.)
- Give leucovorin at 15mg/m2/dose every 6 hours, obtain next level in 6 hours (Hour 36)
  Skip to section 10 (Great work! You have now reached the end of Scenario I. Please ensure that you hit "submit" before moving on.)
- Give leucovorin at 15mg/m2/dose every 6 hours, obtain next level in 12 hours (Hour 42)
  Skip to section 10 (Great work! You have now reached the end of Scenario I. Please ensure that you hit "submit" before moving on.)
- Give leucovorin at 15mg/m2/dose every 6 hours, obtain next level in 18 hours (Hour 48)
  Skip to section 10 (Great work! You have now reached the end of Scenario I. Please ensure that you hit "submit" before moving on.)
- Give leucovorin at 15mg/m2/dose every 6 hours, obtain next level in 24 hours (Hour 54)
  Skip to section 10 (Great work! You have now reached the end of Scenario I. Please ensure that you hit "submit" before moving on.)
- Give leucovorin at 100mg/m2/dose every 6 hours, obtain next level in 6 hours (Hour 36)
  Skip to section 10 (Great work! You have now reached the end of Scenario I. Please ensure that you hit "submit" before moving on.)
- Give leucovorin at 100mg/m2/dose every 6 hours, obtain next level in 12 hours (Hour 42)
  Skip to section 10 (Great work! You have now reached the end of Scenario I. Please ensure that you hit "submit" before moving on.)
- Give leucovorin at 100mg/m2/dose every 6 hours, obtain next level in 18 hours (Hour 48)
  Skip to section 10 (Great work! You have now reached the end of Scenario I. Please ensure that you hit "submit" before moving on.)
- Give leucovorin at 100mg/m2/dose every 6 hours, obtain next level in 24 hours (Hour 54)
  Skip to section 10 (Great work! You have now reached the end of Scenario I. Please ensure that you hit "submit" before moving on.)
- Give glucarpidase 50U/kg/dose IV once
  Skip to section 11 (Great work! You have now reached the end of Scenario I. Please ensure that you hit "submit" before moving on.)
- Discharge the patient home
  Skip to section 12 (Great work! You have now reached the end of Scenario I. Please ensure that you hit "submit" before moving on.)

6. How confident do you feel about your selection? *

Mark only one oval.

	1	2	3	4	5	
I gue	◯	◯	◯	◯	◯	Very confident

At 36 hours, your next set of labs result:

36 hour methotrexate level: 2.5uM
Creatinine: 0.8 mg/dl
Fluid intake over the past 8 hours: 2000cc
Urine output over the past 8 hours: 1650cc
Urine pH: 7.4
Urine specific gravity: 1.006

7. Should fluids be changed? *

Mark only one oval.

◯ No fluid changes are necessary at this time
◯ Increase fluids by 75 ml/m2/hr

8. What is the next best step? *

Mark only one oval.

- ( ) Obtain next level in 6 hours (Hour 42) *Skip to question 10*
- ( ) Obtain next level in 12 hours (Hour 48) *Skip to question 13*
- ( ) Obtain next level in 18 hours (Hour 54) *Skip to question 15*
- ( ) Obtain next level in 24 hours (Hour 60) *Skip to section 9 (Great work! You have now reached the end of Scenario I. Please ensure that you hit "submit" before moving on.)*
- ( ) Give leucovorin at 15mg/m2/dose every 6 hours, obtain next level in 6 hours (Hour 42) *Skip to section 10 (Great work! You have now reached the end of Scenario I. Please ensure that you hit "submit" before moving on.)*
- ( ) Give leucovorin at 15mg/m2/dose every 6 hours, obtain next level in 12 hours (Hour 48) *Skip to section 10 (Great work! You have now reached the end of Scenario I. Please ensure that you hit "submit" before moving on.)*
- ( ) Give leucovorin at 15mg/m2/dose every 6 hours, obtain next level in 18 hours (Hour 54) *Skip to section 10 (Great work! You have now reached the end of Scenario I. Please ensure that you hit "submit" before moving on.)*
- ( ) Give leucovorin at 15mg/m2/dose every 6 hours, obtain next level in 24 hours (Hour 60) *Skip to section 10 (Great work! You have now reached the end of Scenario I. Please ensure that you hit "submit" before moving on.)*
- ( ) Give leucovorin at 100mg/m2/dose every 6 hours, obtain next level in 6 hours (Hour 42) *Skip to section 10 (Great work! You have now reached the end of Scenario I. Please ensure that you hit "submit" before moving on.)*
- ( ) Give leucovorin at 100mg/m2/dose every 6 hours, obtain next level in 12 hours (Hour 48) *Skip to section 10 (Great work! You have now reached the end of Scenario I. Please ensure that you hit "submit" before moving on.)*
- ( ) Give leucovorin at 100mg/m2/dose every 6 hours, obtain next level in 18 hours (Hour 54) *Skip to section 10 (Great work! You have now reached the end of Scenario I. Please ensure that you hit "submit" before moving on.)*
- ( ) Give leucovorin at 100mg/m2/dose every 6 hours, obtain next level in 24 hours (Hour 60) *Skip to section 10 (Great work! You have now reached the end of Scenario I. Please ensure that you hit "submit" before moving on.)*
- ( ) Give glucarpidase 50U/kg/dose IV once *Skip to section 11 (Great work! You have now reached the end of Scenario I. Please ensure that you hit "submit" before moving on.)*
- ( ) Discharge the patient home *Skip to section 12 (Great work! You have now reached the end of Scenario I. Please ensure that you hit "submit" before moving on.)*

9. How confident do you feel about your selection? *

Mark only one oval.

	1	2	3	4	5	
I gue	( )	( )	( )	( )	( )	Very confident

Your next set of labs result:

42 hour methotrexate level: 1.9uM
Creatinine: 0.7 mg/dl
Fluid intake over the past 8 hours: 2200cc
Urine output over the past 8 hours: 1950cc
Urine pH: 7.1
Urine specific gravity: 1.011

10. Should fluids be changed? *

Mark only one oval.

- No fluid changes are necessary at this time
- Increase fluids by 75 ml/m2/hr

11. What is the next best step? *

Mark only one oval.

- Obtain next level in 6 hours (Hour 48) *Skip to question 13*
- Obtain next level in 12 hours (Hour 54) *Skip to question 15*
- Obtain next level in 18 hours (Hour 60) *Skip to question 17*
- Obtain next level in 24 hours (Hour 66)
  Skip to section 9 (Great work! You have now reached the end of Scenario I. Please ensure that you hit “submit” before moving on.)
- Give leucovorin at 15mg/m2/dose every 6 hours, obtain next level in 6 hours (Hour 48) *Skip to question 13*
- Give leucovorin at 15mg/m2/dose every 6 hours, obtain next level in 12 hours (Hour 54) *Skip to question 15*
- Give leucovorin at 15mg/m2/dose every 6 hours, obtain next level in 18 hours (Hour 60) *Skip to question 17*
- Give leucovorin at 15mg/m2/dose every 6 hours, obtain next level in 24 hours (Hour 66)
  Skip to section 9 (Great work! You have now reached the end of Scenario I. Please ensure that you hit “submit” before moving on.)
- Give leucovorin at 100mg/m2/dose every 6 hours, obtain next level in 6 hours (Hour 48)
  Skip to section 13 (Great work! You have now reached the end of Scenario I. Please ensure that you hit “submit” before moving on.)
- Give leucovorin at 100mg/m2/dose every 6 hours, obtain next level in 12 hours (Hour 54)
  Skip to section 13 (Great work! You have now reached the end of Scenario I. Please ensure that you hit “submit” before moving on.)
- Give leucovorin at 100mg/m2/dose every 6 hours, obtain next level in 18 hours (Hour 60)
  Skip to section 13 (Great work! You have now reached the end of Scenario I. Please ensure that you hit “submit” before moving on.)
- Give leucovorin at 100mg/m2/dose every 6 hours, obtain next level in 24 hours (Hour 66)
  Skip to section 13 (Great work! You have now reached the end of Scenario I. Please ensure that you hit “submit” before moving on.)
- Give glucarpidase 50U/kg/dose IV once
  Skip to section 11 (Great work! You have now reached the end of Scenario I. Please ensure that you hit “submit” before moving on.)
- Discharge the patient home
  Skip to section 12 (Great work! You have now reached the end of Scenario I. Please ensure that you hit “submit” before moving on.)

12. How confident do you feel about your selection? *

Mark only one oval.

	1	2	3	4	5	
I gue	◯	◯	◯	◯	◯	Very confident

Your next set of labs result:

48 hour methotrexate level: 0.5uM
Creatinine: 0.8 mg/dl
Fluid intake over the past 8 hours: 2000cc
Urine output over the past 8 hours: 1700cc
Urine pH: 7.3
Urine specific gravity: 1.009

13. Should fluids be changed? *

Mark only one oval.

◯ No fluid changes are necessary at this time

◯ Increase fluids by 75 ml/m2/hr

14. What is the next best step? *

Mark only one oval.

- Obtain next level in 6 hours (Hour 54) *Skip to question 15*
- Obtain next level in 12 hours (Hour 60) *Skip to question 17*
- Obtain next level in 18 hours (Hour 66)
  Skip to section 9 (Great work! You have now reached the end of Scenario I. Please ensure that you hit "submit" before moving on.)
- Obtain next level in 24 hours (Hour 72)
  Skip to section 9 (Great work! You have now reached the end of Scenario I. Please ensure that you hit "submit" before moving on.)
- Give leucovorin at 15mg/m2/dose every 6 hours, obtain next level in 6 hours (Hour 54) *Skip to question 15*
- Give leucovorin at 15mg/m2/dose every 6 hours, obtain next level in 12 hours (Hour 60) *Skip to question 17*
- Give leucovorin at 15mg/m2/dose every 6 hours, obtain next level in 18 hours (Hour 66)
  Skip to section 9 (Great work! You have now reached the end of Scenario I. Please ensure that you hit "submit" before moving on.)
- Give leucovorin at 15mg/m2/dose every 6 hours, obtain next level in 24 hours (Hour 72)
  Skip to section 9 (Great work! You have now reached the end of Scenario I. Please ensure that you hit "submit" before moving on.)
- Give leucovorin at 100mg/m2/dose every 6 hours, obtain next level in 6 hours (Hour 54)
  Skip to section 13 (Great work! You have now reached the end of Scenario I. Please ensure that you hit "submit" before moving on.)
- Give leucovorin at 100mg/m2/dose every 6 hours, obtain next level in 12 hours (Hour 60)
  Skip to section 13 (Great work! You have now reached the end of Scenario I. Please ensure that you hit "submit" before moving on.)
- Give leucovorin at 100mg/m2/dose every 6 hours, obtain next level in 18 hours (Hour 66)
  Skip to section 13 (Great work! You have now reached the end of Scenario I. Please ensure that you hit "submit" before moving on.)
- Give leucovorin at 100mg/m2/dose every 6 hours, obtain next level in 24 hours (Hour 72)
  Skip to section 13 (Great work! You have now reached the end of Scenario I. Please ensure that you hit "submit" before moving on.)
- Give glucarpidase 50U/kg/dose IV once
  Skip to section 11 (Great work! You have now reached the end of Scenario I. Please ensure that you hit "submit" before moving on.)
- Discharge the patient home
  Skip to section 12 (Great work! You have now reached the end of Scenario I. Please ensure that you hit "submit" before moving on.)

Your next set of labs result:

54 hour methotrexate level: 1.1uM
Creatinine: 0.6 mg/dl
Fluid intake over the past 8 hours: 2050cc
Urine output over the past 8 hours: 2100cc
Urine pH: 7.1
Urine specific gravity: 1.008

15. Should fluids be changed? *

Mark only one oval.

- No fluid changes are necessary at this time
- Increase fluids by 75 ml/m2/hr

16. What is the next best step? *

Mark only one oval.

- Obtain next level in 6 hours (Hour 60) *Skip to question 17*
- Obtain next level in 12 hours (Hour 66)
  Skip to section 9 (Great work! You have now reached the end of Scenario I. Please ensure that you hit "submit" before moving on.)
- Obtain next level in 18 hours (Hour 72)
  Skip to section 9 (Great work! You have now reached the end of Scenario I. Please ensure that you hit "submit" before moving on.)
- Obtain next level in 24 hours (Hour 78)
  Skip to section 9 (Great work! You have now reached the end of Scenario I. Please ensure that you hit "submit" before moving on.)
- Give leucovorin at 15mg/m2/dose every 6 hours, obtain next level in 6 hours (Hour 60) *Skip to question 17*
- Give leucovorin at 15mg/m2/dose every 6 hours, obtain next level in 12 hours (Hour 66)
  Skip to section 9 (Great work! You have now reached the end of Scenario I. Please ensure that you hit "submit" before moving on.)
- Give leucovorin at 15mg/m2/dose every 6 hours, obtain next level in 18 hours (Hour 72)
  Skip to section 9 (Great work! You have now reached the end of Scenario I. Please ensure that you hit "submit" before moving on.)
- Give leucovorin at 15mg/m2/dose every 6 hours, obtain next level in 24 hours (Hour 78)
  Skip to section 9 (Great work! You have now reached the end of Scenario I. Please ensure that you hit "submit" before moving on.)
- Give leucovorin at 100mg/m2/dose every 6 hours, obtain next level in 6 hours (Hour 60)
  Skip to section 13 (Great work! You have now reached the end of Scenario I. Please ensure that you hit "submit" before moving on.)
- Give leucovorin at 100mg/m2/dose every 6 hours, obtain next level in 12 hours (Hour 66)
  Skip to section 13 (Great work! You have now reached the end of Scenario I. Please ensure that you hit "submit" before moving on.)
- Give leucovorin at 100mg/m2/dose every 6 hours, obtain next level in 18 hours (Hour 72)
  Skip to section 13 (Great work! You have now reached the end of Scenario I. Please ensure that you hit "submit" before moving on.)
- Give leucovorin at 100mg/m2/dose every 6 hours, obtain next level in 24 hours (Hour 78)
  Skip to section 13 (Great work! You have now reached the end of Scenario I. Please ensure that you hit "submit" before moving on.)
- Give glucarpidase 50U/kg/dose IV once
  Skip to section 11 (Great work! You have now reached the end of Scenario I. Please ensure that you hit "submit" before moving on.)
- Discharge the patient home
  Skip to section 12 (Great work! You have now reached the end of Scenario I. Please ensure that you hit "submit" before moving on.)

Your next set of labs result:

60 hour methotrexate level: 0.08uM
Creatinine: 0.8 mg/dl
Fluid intake over the past 8 hours:
Urine output over the past 8 hours:
Urine pH: 7.0
Urine specific gravity: 1.011

17. Should fluids be changed? *

Mark only one oval.

- No fluid changes are necessary at this time
- Increase fluids by 75 ml/m2/hr

18. What is the next best step? *

Mark only one oval.

- ◯ Obtain next level in 6 hours (Hour 66)
  Skip to section 14 (Great work! You have now reached the end of Scenario I. Please ensure that you hit “submit” before moving on.)
- ◯ Obtain next level in 12 hours (Hour 72)
  Skip to section 14 (Great work! You have now reached the end of Scenario I. Please ensure that you hit “submit” before moving on.)
- ◯ Obtain next level in 18 hours (Hour 78)
  Skip to section 14 (Great work! You have now reached the end of Scenario I. Please ensure that you hit “submit” before moving on.)
- ◯ Obtain next level in 24 hours (Hour 84)
  Skip to section 14 (Great work! You have now reached the end of Scenario I. Please ensure that you hit “submit” before moving on.)
- ◯ Give leucovorin at 15mg/m2/dose every 6 hours, obtain next level in 6 hours (Hour 66)
  Skip to section 14 (Great work! You have now reached the end of Scenario I. Please ensure that you hit “submit” before moving on.)
- ◯ Give leucovorin at 15mg/m2/dose every 6 hours, obtain next level in 12 hours (Hour 72)
  Skip to section 14 (Great work! You have now reached the end of Scenario I. Please ensure that you hit “submit” before moving on.)
- ◯ Give leucovorin at 15mg/m2/dose every 6 hours, obtain next level in 18 hours (Hour 78)
  Skip to section 14 (Great work! You have now reached the end of Scenario I. Please ensure that you hit “submit” before moving on.)
- ◯ Give leucovorin at 15mg/m2/dose every 6 hours, obtain next level in 24 hours (Hour 84)
  Skip to section 14 (Great work! You have now reached the end of Scenario I. Please ensure that you hit “submit” before moving on.)
- ◯ Give leucovorin at 100mg/m2/dose every 6 hours, obtain next level in 6 hours (Hour 66)
  Skip to section 14 (Great work! You have now reached the end of Scenario I. Please ensure that you hit “submit” before moving on.)
- ◯ Give leucovorin at 100mg/m2/dose every 6 hours, obtain next level in 12 hours (Hour 72)
  Skip to section 14 (Great work! You have now reached the end of Scenario I. Please ensure that you hit “submit” before moving on.)
- ◯ Give leucovorin at 100mg/m2/dose every 6 hours, obtain next level in 18 hours (Hour 78)
  Skip to section 14 (Great work! You have now reached the end of Scenario I. Please ensure that you hit “submit” before moving on.)
- ◯ Give leucovorin at 100mg/m2/dose every 6 hours, obtain next level in 24 hours (Hour 84)
  Skip to section 14 (Great work! You have now reached the end of Scenario I. Please ensure that you hit “submit” before moving on.)
- ◯ Give glucarpidase 50U/kg/dose IV once
  Skip to section 11 (Great work! You have now reached the end of Scenario I. Please ensure that you hit “submit” before moving on.)
- ◯ Discharge the patient home
  Skip to section 15 (Great work! You have now reached the end of Scenario I. Please ensure that you hit “submit” before moving on.)

Great work! You have now reached the end of Scenario I. Please ensure that you hit “submit” before moving on.

Great work! You have now reached the end of Scenario I. Please ensure that you hit “submit” before moving on.

Great work! You have now reached the end of Scenario I. Please ensure that you hit “submit” before moving on.

Great work! You have now reached the end of Scenario I. Please ensure that you hit “submit” before moving on.

Great work! You have now reached the end of Scenario I. Please ensure that you hit “submit” before moving on.

Great work! You have now reached the end of Scenario I. Please ensure that you hit “submit” before moving on.

Great work! You have now reached the end of Scenario I. Please ensure that you hit “submit” before moving on.

This content is neither created nor endorsed by Google.

Google Forms
